# Pigment Epithelium Derived Factor Is Involved in the Late Phase of Osteosarcoma Metastasis by Increasing Extravasation and Cell-Cell Adhesion

**DOI:** 10.3389/fonc.2022.818182

**Published:** 2022-01-31

**Authors:** Sei Kuriyama, Gentaro Tanaka, Kurara Takagane, Go Itoh, Masamitsu Tanaka

**Affiliations:** ^1^ Department of Molecular Medicine and Biochemistry, Graduate School and Faculty of Medicine, Akita University, Akita City, Japan; ^2^ Department of Lifescience, Faculty and Graduate School of Engineering and Resource Science, Akita University, Akita City, Japan

**Keywords:** osteosarcoma, pigment epithelium derived factor (PEDF), metastasis, mesenchymal to epithelial transition (MET), extravasation

## Abstract

Organ tropism of metastatic cells is not well understood. To determine the key factors involved in the selection of a specific organ upon metastasis, we established metastatic cell lines and analyzed their homing to specific tissues. Toward this, 143B osteosarcoma cells were injected intracardially until the kidney-metastasizing sub-cell line Bkid was established, which significantly differed from the parental 143B cells. The candidate genes responsible for kidney metastasis were validated, and SerpinF1/Pigment epithelium derived factor (PEDF) was identified as the primary target. Bkid cells with PEDF knockdown injected intracardially did not metastasize to the kidneys. In contrast, PEDF overexpressing 143B cells injected into femur metastasized to the lungs and kidneys. PEDF triggered mesenchymal-to-epithelial transition (MET) *in vitro* as well as *in vivo*. Based on these results, we hypothesized that the MET might be a potential barrier to extravasation. PEDF overexpression in various osteosarcoma cell lines increased their extravasation to the kidneys and lungs. Moreover, when cultured close to the renal endothelial cell line TKD2, Bkid cells disturbed the TKD2 layer and hindered wound healing *via* the PEDF-laminin receptor (lamR) axis. Furthermore, novel interactions were observed among PEDF, lamR, lysyl oxidase-like 1 (Loxl1), and SNAI3 (Snail-like transcription factor) during endothelial-to-mesenchymal transition (EndoMT). Collectively, our results show that PEDF induces cancer cell extravasation by increasing the permeability of kidney and lung vasculature acting *via* lamR and its downstream genes. We also speculate that PEDF promotes extravasation *via* inhibiting EndoMT, and this warrants investigation in future studies.

## Introduction

Despite being a rare type of cancer, osteosarcoma (OS) is the most common bone cancer, and occurs mainly in children and young adults. Most of the currently used surgical treatments that are combined with multiple-agent chemotherapy had been established during the 70s–80s. The survival rate of OS patients had improved at that time; however, further significant treatments have not been developed (1, 2). OS has heterogenous characteristics, and lacks any consistent unifying event that could lead to its pathogenesis (1). High-grade osteosarcomas have a high propensity for pulmonary metastasis; over 75% of metastatic OS cases involve the lung. Other organ metastases are extremely rare; however, the renal metastasis of OS is often found with pulmonary metastasis after death (3), and the hepatic metastasis of OS is found microscopically after chemotherapy (4). Usually the OS metastasize hematogeneously to lung and bones but rarely to lymph nodes (5). It is crucial to determine the mechanism by which osteosarcomas prefer to metastasize to specific organs, as may be applicable to other cancers. However, most established OS cell lines were found to be non-metastatic.

A v-Ki-ras-transformed cell line derived from the human osteosarcoma (HOS) cell line 143B showed better growth and survival in mice than HOS. It has been reported that 143B cells form pulmonary metastases from orthotopic transplantations into the tibia (6–8), even from a cutaneous tumor (9). Therefore, we designed an experiment in which the orthotopic implantation of 143B caused distant metastatic lesions in the various organs. However, our 143B cells did not show such strong phenotypes from the knee joint injection or subcutaneous injection (data not shown). We then tried to examine the intracardiac injection to mimic circulating OS. In the orthotopic experimental model, OS partially digests the bone, which may provide TGF-ß, subsequently experiences neovascularization, intravasation, migration toward the distant organ, anoikis resistance, and extravasation. Finally, OS proliferates in the secondary site (10). However, OS can start as the circulating population in the intracardiac injection model. Therefore, the key function would be to elucidate the mechanism of OS survival, arrest on a particular organ, extravasation, and proliferation under suitable conditions. Anoikis resistance and anchoring on the particular sites of the endothelial cells could be mediated by various molecules, but it is ultimately consolidated into the Akt pathway (10). Additionally, SDF1-CXCR4 could be involved in the lung preference of OS metastasis (11, 12). However, the cell surface anchor between OS and endothelial cells remains unclear.

We successfully created metastatic sub-cell lines, and identified the genes potentially responsible for the specificity of metastasized organ. The details are presented in the following result section. Furthermore, we identified SERPINF1/pigment epithelium-derived factor (PEDF) as the gene responsible for the renal metastasis from the established renal metastatic cell line, Bkid.

PEDF is a Serine protease inhibitor family protein displaying no inhibitory activity against serine protease (13). PEDF has strong anti-angiogenesis activity, and its signaling is mediated by laminin receptor (lamR), which is also known as RPSA (40S ribosomal protein SA) (14, 15). PEDF is multifunctional, and has another PEDF receptor, which is also known as PNPLA2, and is a membrane-bound lipase molecule (16, 17). PEDF has been reported to have anti-angiogenesis, anti-tumor, and anti-metastasis properties in cancer (18–20). Particularly in OS, PEDF was tested using SaOS-2, and it was found that PEDF overexpression reduced the volume of the tumor and microvessels in a mouse model (20). Furthermore, the administration of recombinant PEDF protein in the orthotopic spontaneous metastasis model using SaOS-2 showed therapeutic effects in the primary and secondary OS lesions (21, 22). However, a recent study reported that the role of endogenous PEDF in cancer metastasis remains controversial and dependent on the context of cancer cell types (23). Furthermore, endogenous PEDF expression was observed in almost all osteosarcomas (**Supplemental Figure 1A**). Therefore, we hypothesized that endogenous PEDF may play an unknown role in cancer metastasis.

In the present study, loss of PEDF function in Bkid tumors blocked renal metastasis, and gain-of-function exhibited pulmonary and renal metastasis. We also found that PEDF expression increased the potency of mesenchymal-to-epithelial transition (MET); however, we expected that MET might block extravasation before forming nodules in the organs. Contrary to this expectation, PEDF overexpression increased extravasation in various osteosarcomas. Therefore, we examined the mechanism underlying the extravasation. As it was expressed in the kidneys and lungs, we further investigated the downstream signaling of lamR, which is one of the receptor of PEDF (14) in the renal endothelial cell line TKD2. As a result, lysyl oxidase-like 1 (Loxl1) (24) and SNAI3 (25) worked through the PEDF-lamR axis in TKD2, and we elucidated that their direct molecular interactions, while Snail2 and the other EMT/MET factors were regulated in cancer. Collectively, these results suggest that PEDF might increase extravasation, which is controlled by endogenous receptor expression. Therefore, it exhibited organ specificity, and extravasated cancer cells may undergo MET to form secondary lesions. This study demonstrated a new role for endogenous PEDF, particularly in the late phase of metastasis.

## Materials and Methods

### Cell Lines and Culture Methods

We obtained 143B cells from the RIKEN BRC Cell Bank (Cat# RCB0701, RRID: CVCL_9W36). Other osteosarcoma cell lines were obtained from our collaborator (26). The osteosarcoma cells were maintained in Eagle’s modified essential medium (EMEM) (Millipore Sigma, Burlington, MA, USA) with 10% fetal bovine serum (FBS) and sodium pyruvate at 37°C and 5% CO_2_. The modified or subcloned cell lines of 143B and the other osteosarcoma cell lines with lentivirus/retrovirus constructs (see **Supplemental Text**) were also cultured in EMEM+10% FBS along with the selection antibiotic G418 sulfate (800 µg/mL, Inalco Pharmaceuticals, CA, USA), puromycin (2 µg/mL), or hygromycin B (400 µg/mL, Wako-Fujifilm, Osaka, Japan). Mouse kidney endothelial TKD2 cells (RCB Cat# RCB0752, RRID: CVCL_5598) were maintained in Dulbecco’s modified essential medium (DMEM) containing 4500 mg/mL L-glucose, 2% FBS, sodium pyruvate, ITS-X supplement (x1 from x100 stock solution, Wako-Fujifilm), and 2.5 µg/mL EGF at 33°C, and 5% CO_2_.

### Lentivirus and Retrovirus Preparation

A modified CSII-CMV-vector with a puromycin-resistant gene was used to establish the 143B cell line, as described previously (27). The selection of the cells with antibiotics and the information on the vector are described in the **Supplemental Text**.

### Animal Experiments and *In Vivo* Imaging

All animal experiments were approved by the Akita University Ethical Committee for Experimental Animals. The 143B cells were cultured until subconfluent, rinsed with PBS, resuspended in Hank’s buffered saline (HBSS), and injected into the left cardiac ventricle of nude mice (Balb/c Slc, SLC JAPAN, five-weeks-old) using 31G needle syringes containing 4 × 10^5^ cells/100 µL. The tumors were monitored once per week by administering *aLuciferin* (250 µL/25 g nude mouse, 15 mg/mL in PBS) (Avidin Ltd, Szeged, Hungary) and were observed using the IVIS imaging suite (IVIS Lumina, Perkin-Elmer, Waltham, MA, USA). In a separate experiment, mice were anesthetized using isoflurane vapor, and 5 × 10^4^ cells, 2 × 10^6^ cells in 10 µL were injected into their knee joints using a Hamilton syringe.

### Microarray Analyses

RNA was obtained from established cell lines, and gene expression was analyzed using a Gene Chip (Human v.2.0, GE Healthcare, Chicago, IL, USA). Gene expression analyses and drawing of the graphs or charts were performed using Transcriptome Analysis Console 4.0 software (Affymetrix Transcriptome Analysis Console Software, RRID : SCR_018718).

### RNA Extraction, cDNA Synthesis, and Quantitative Polymerase Chain Reaction

The cells were cultured under the appropriate conditions for each experiment. RNA was extracted using PureLink RNA Mini Kit (Thermo Fisher Scientific, Waltham, MA, USA). cDNA was synthesized using the PrimeScript 1st strand cDNA Synthesis Kit (Takara Bio Inc., Shiga, JAPAN). Quantitative PCR was performed on a LightCycler Nano system (Roche Molecular systems Inc, Pleasanton, CA, USA) using Brilliant III Ultra-Fast SYBR Green master mix (Agilent Technologies, Santa Clara, CA, USA). The sequential steps of reverse transcript and quantitative PCR will be referred as qRT-PCR.

### Oligonucleotides

All qRT-PCR primers and oligonucleotides used for gene editing are listed in the **Supplemental Text**.

The guide RNAs were transcribed using an all-in-one CRISPR/Cas9 system (Thermo Fisher Scientific). Human and mouse laminin receptor genome sequences were edited using the PITCh method (Precise Integration into Target Chromosome) (28, 29). The target sequences were also listed in **Supplemental Data**.

### Western Blots and Antibodies

The cells were lysed using PLC buffer (100 mM Tris-HCl, 1% TritonX-100, 10% glycerol, 50 mM sodium vanadate, 1 mM PMSF, and 1 × protease inhibitor cocktail). Before obtaining their organs, the mice were perfused with PBS to remove blood from the organs. Each mouse organ was dissected briefly using scissors and homogenized in PLC buffer. Large fragments were removed by centrifugation, and the cleared samples were treated as described above. The antibodies used in this study were follows: Anti-PEDF/SerpinF1 (R&D Systems Cat# AF1177, RRID : AB_2187173), Anti-Cadherin-11 (R&D Systems Cat# MAB1790, RRID : AB_2076970), Anti-N-cadherin (BD Biosciences Cat# 610920, RRID : AB_2077527), Anti-K-cadherin (Millipore Cat# MAB2013, RRID : AB_11210468), Anti-CD31 (Abcam Cat# ab28364, RRID : AB_72636), Anti-SPARC (Sigma-Aldrich Cat# HPA003020, RRID : AB_1079531), Anti-laminin receptor (RPSA/67LR) (GeneTex Cat# GTX23099, RRID : AB_367077), Anti-PNPLA2 (R&D Systems Cat# AF5365, RRID : AB_2165678), Anti-phosphor-Akt (Ser473(D9E)XP, Cell Signaling Technology Cat# 5012, RRID : AB_2224726), Anti-Akt (CST, Cat# 9272, RRID : AB_329827), Anti-phospho p38 (CST, Cat# 9210, RRID : AB_330710), Anti-p38 MAPK (CST, Cat# 9212, RRID : AB_330713), Anti-phospho p42/44 MAPK (CST, Cat# 4094, RRID : AB_10694057), Anti-p42/44 MAPK (CST, Cat# 9102, RRID : AB_330744), Anti-Slug (Snail2) (CST, Cat# 9585, RRID : AB_2239535), Anti-MMP9 (Millipore Cat# AB19016, RRID : AB_91090), Anti-MMP14 (Abcam Cat# ab51074, RRID : AB_881234), Anti-MMP15 (Abcam Cat# ab15475, RRID : AB_301885), Anti-Lipoma preferred partner (LPP) (Abcam Cat# ab63621, RRID : AB_956113) Anti-ß-catenin (Abcam Cat# ab79089, RRID : AB_1603423), Anti-alpha-tubulin (Sigma-Aldrich Cat# T5168, RRID : AB_477579).

### Immunohistochemistry (IHC) and Immunofluorescence (IF)

The procedures of immunostaining have been described previously (30). The preparation of the paraffin-embedded samples and sectioning of the paraffin blocks were provided by Akita University Bioscience Education-Research Support Center.

### Miles Assay

We followed the procedure described in the text and video of a previous study (31). The stock solution (5%) of Evans blue (EB) (Wako-Fujifilm) was prepared in PBS, and the diluted (0.5%) solution was sterilized using a 0.45 µm syringe filter (SARSTEDT AG&Co.KG, Nümbrecht, Germany). EB was introduced through the tail vein of mice injected with MG63 cells two weeks ago. After each organ was dissected, the weights of each organ were measured, and the EB in each organ was extracted using formamide solution for 24 h. The absorbance of EB was measured at 610 nm using a Multiskan FC system (Thermo Fisher Scientific).

### Double-Sided Culture of Cancer Cluster and the Endothelial Layer

GFP-expressing TKD2 (TKD2-GFP) cell line was established. The TKD2-GFP cells were cultured on the bottom side of the culture insert by flipping the atelocollagen membrane insert (AteloCell CM-24, Koken, Japan). Next, 143B and 143B+PEDF cells were separately cultured on low-adherent culture dishes for 2 days, and 143B/143B+PEDF spheroids were prepared. After obtaining the confluent layer of TKD2-GFP cells, the culture insert was flipped again, and a few spheroids of 143B/143B+PEDF were placed on the opposite side of the membrane over the TKD2-GFP cell layer (see schematic diagram in **Figure 6E**). After 2 days, the culture inserts were briefly fixed with 4% PFA, washed and permealized by PBS+0.01% TritonX-100, and immuno-stained with anti-ß-catenin antibody. Next, the membranes were cut out from the culture insert, and the samples were mounted on a slide with Mowiol (Sigma). The images were obtained under the confocal microscope LSM780 (Carl Zeiss Microscopy GmbH, Jena, Germany), and the Z-stack images were reconstructed by IMARIS software (Imaris, RRID : SCR_007370).

### Cell Proliferation and Wound Healing Assay

To obtain data on cell proliferation and wound healing assays, we performed experiments on the Holomonitor M4 (HoloMonitor system, RRID : SCR_019231). For cell proliferation counting, six-well plates were used (SARSTEDT), the multiple positions were imaged, and the cell numbers were analyzed using Hstudio software (version 2.7.3, PHI AB). For wound healing assay of TKD2, we used the culture-insert two-well plate to separate the monolayer of the culture cells (ibidi #80206, Martinsrid, Germany). The cell-free area of the wound was imaged, and the reduction in the area was also analyzed using Hstudio software.

### Immunoprecipitation and HaloTag Pulldown

For FLAG-tag immunoprecipitation (IP), cells were lysed using RIPA buffer (50mM TrisHCl, 150mM NaCl, 0.5% Sodium Deoxycholate, 0.1% Sodium dodecyl sulfate, 1% NP-40). The input was taken before IP. FLAG-M2 affinity agarose gels (Sigma, A2220) were washed by RIPA buffer several times until washing solution became clear, blocked using 0.1% BSA in RIPA buffer for 30 minutes, washed once, and mixed with 0.5 mg/mL protein lysates for 3 hours at 4°C. The agarose bed was washed using RIPA buffer three times for 5 minute at 4°C. The washed agarose bed was incubated with 100µg/mL of 3xFLAG peptides (Sigma) in TBST buffer for 1 hour. The eluates were separated from the bed by centrifuge. For HaloTag purification, cells were lysed using HaloTag purification buffer (50mM HEPES, 1mM Dithiothreitol, 1mM EDTA, 1mM EGTA, 0.05% NP-40). HaloTag acryl beads were rinsed twice with HaloTag purification buffer, and mixed with 0.5 mg/mL protein lysate for 30 minutes at room temperature. The beads were washed with HaloTag purification buffer three times for 5 minutes at room temperature. The beads was boiled for 3 minutes to extract the proteins.

## Results

### Establishment of the Sub-Cell Lines From the Intracardiac Injections of 143B

**Figure 1:**
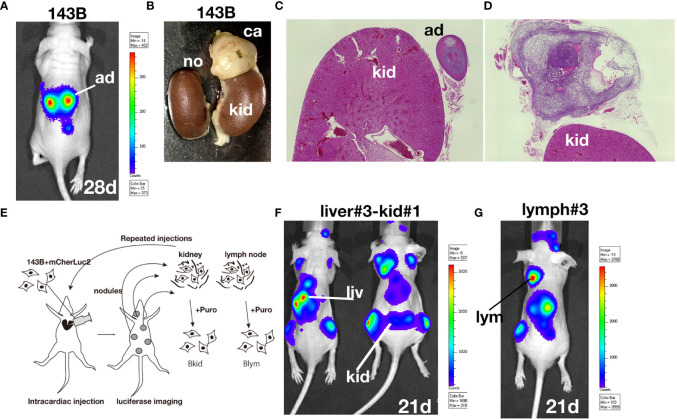
Establishment of osteosarcoma sub-cell lines. **(A)** *In vivo* luciferase imaging of 143B expressing mCherry-IRES-luciferase 2 (143B) after four weeks. Signals in the adrenal glands were detected after two–three weeks. **(B)** Kidneys dissected with the adrenal gland of 143B-injected mice. **(C)** There was no renal metastatic lesion on the left side of **(B)**. **(D)** Cancer cells infiltrated the adrenal gland, while no renal metastasis was observed. **(E)** Schematic diagram of repeated intracardiac injections of osteosarcoma cells. **(F, G)** Results of intracardiac injections of the aggressive cell line. **(F)** The lesions quickly spread throughout the nude mice within three weeks. The cells metastasized to the liver three times and started to infiltrate the kidney. Note that the renal signals of luciferase appeared before the adrenal gland signal became dense. **(G)** Metastatic lesions in the shoulder lymph node after two-repeated injections gathered from lymph node metastasis. The luciferase signal in the lymph node was higher than that in the adrenal glands.

According to the previous paper, 143B, an osteosarcoma cell line, could metastasize into lung even from the cutaneous tumor (9). We were especially interested in the highly metastatic potential of 143B; therefore, we prepared our own 143B +mCherry-IRES-luciferase2 sub-cell line (referred to as 143B). However, 143B did not show pulmonary metastasis for four–six weeks, both from orthotopic or subcutaneous injection (data not shown). Therefore, we performed intracardiac injection of 143B; the cells were monitored by luciferase imaging and were soon observed in the adrenal glands (**Figures 1A–D**). We further followed the secondary metastatic lesions (data not shown) and enhanced the organ-tropism of metastasis by repeated injections to establish different sub-cell lines (**Figure 1E**). Once 143B cells consistently metastasized into the liver (called Bliv), they were used for several repeated injections, following which the cells were suddenly biased to renal metastasis (**Figure 1F**) or lymph node metastasis (**Figure 1G**). The initial metastasis in the specific organ was observed very weakly after four to five weeks from the cardiac injection, however, the repeated injections increased the aggressiveness of these sub-cell lines, and the luciferase signals could be observed within three weeks after the third time injection since the sub-population was detected in the specific organs (data not shown). Finally, we established two biased metastatic sub-cell lines from 143B: the renal-specific Bkid and lymph node-specific Blym.

### PEDF Is Upregulated in Sub-Cell Lines That Frequently Infiltrate into the Kidney

**Figure 2:**
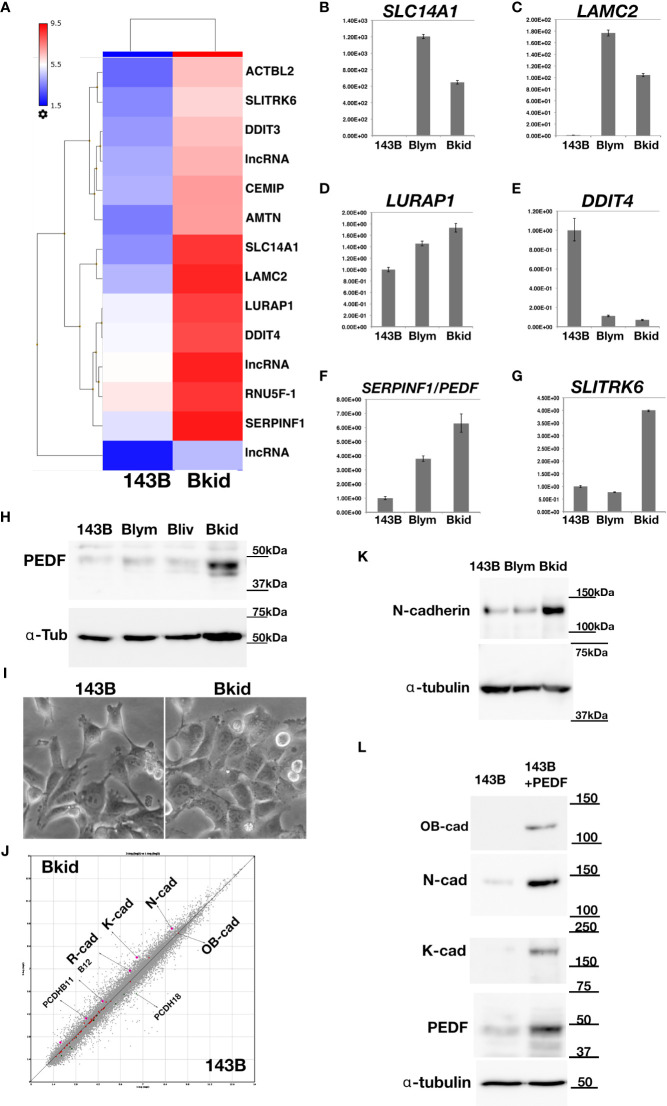
Gene expression profile between 143B parental (143B) and 143B renal metastatic cells (Bkid). **(A)** The hierarchy plot of the gene expression profiles of the comparison between 143B and Bkid. **(B–G)** The validations of gene-tip analyses by quantitative RT-PCR (qRT-PCR). The results of qPCR with the primer sets: **(B)** *SLC14A1*, urea transporter gene **(C)** laminin C2 chain gene (*LAMC2*). **(D)** *LURAP1* **(E)** *DDIT4* **(F)** *Serin protease inhibitor F1*/*pigment epithelium derived factor* (*PEDF*) **(G)** *Slit related kinase 6* (*Slitrk6*) **(H)** Immunoblot by anti-PEDF antibody or anti-alpha tubulin among sub-cell lines. Blym is a cell line from lesion of lymph node. Bliv is a cell line from hepatic metastasis, later developed as Bkid. **(I)** The morphological changes between 143B and Bkid cells. **(J)** The scatter plot of the gene expression profiles of cadherin family genes between 143B and Bkid. Cdh6 (K-cadherin), Cdh2 (N-cadherin) and Cdh4 (R-cadherin) expression levels were upregulated. **(K)** The endogenous N-cadherin increased in Bkid cells. **(L)** PEDF overexpression in 143B reproduced Bkid phenotypes. OB-cad, N-cad, and K-cad were increased co-incidentally with PEDF overexpression.

After establishing the sub-cell lines of 143B, we performed gene chip (Human Gene 2.0 ST array, Affymetrix) analyses of 143B +mCherryIRES-luc2 (143B), Bkid (renal metastasis), and Blym (lymph node metastasis). A total of 48226 transcript clusters (tc) were analyzed; 523 tc (> 2.0-fold increase) was found in the comparison between Bkid and 143B, whereas 273 tc (> 2.0-fold increase) was found in the comparison between Blym and 143B. Therefore, Bkid was considered the most significantly different subclone of 143B. We then selected genes that were increased in Bkid compared to 143B. The results are summarized in **Table 1**. A hierarchy plot between 143B and Bkid revealed the most effectively upregulated genes in Bkid (**Figure 2A**). We further validated these results using quantitative real time polymerase chain reaction (qRT-PCR) (**Figures 2B–G**). The levels of SLC14A1 (32), LAMC2 (33), LURAP1 (34), and DDIT4 (35) were slightly different from the results of the microarray analyses (**Figures 2A–D**). Serin proteinase inhibitor family F1/Pigment epithelium derived factor (SERPINF1/PEDF) (36), slit-related kinase 6 (Slitrk6) (37), and Amelotin (38) (data not shown) remained as candidates (**Figures 2F, G**). Further validation of the gene expression profiles of these upregulated genes showed that PEDF was expressed in various osteosarcoma cell lines (**Supplementary Figure 1A**), whereas Slitrk6 protein was truncated in the SaOS-2 cell line (**Supplementary Figure 1B**). Bkid cells were derived from the hepatic metastasized sub-cell line (Bliv), as described above. Thus, we examined whether PEDF was acquired by 143B cells (**Figure 2H**). Immunoblot analysis revealed that PEDF did not increase in Bliv and accumulated after Bkid differentiation (**Figure 2H**). Thus, PEDF was considered the best candidate gene for kidney-specific metastasis in 143B cells.

**Table 1:** Gene expression comparison between 143B and Bkid cells.

ID	Bkid Avg	143B Avg	Fold Change	Gene Symbol	Description
16852179	8.68	3.75	30.51	SLC14A1	solute carrier family 14 (urea transporter),
16674845	8.94	4.36	23.76	LAMC2	laminin, gamma 2
16829570	9.13	5.03	17.16	SERPINF1	pigment epithelium derived factor), member 1
16967513	7.04	3.45	12.07	AMTN	amelotin
16670185	9.03	5.55	11.15	LOC284561	uncharacterized LOC284561
16996415	6.51	3.14	10.38	ACTBL2	actin, beta-like 2
17083614	8.52	5.3	9.37	LURAP1L	leucine rich adaptor protein 1-like
16705961	8.34	5.38	7.79	DDIT4	DNA damage inducible transcript 4
16803754	7.08	4.29	6.93	CEMIP	cell migration inducing protein
16686271	8.68	5.89	6.92	RNU5F-1	RNA, U5F small nuclear 1
16766578	6.52	3.9	6.15	DDIT3	DNA-damage-inducible transcript 3
16942367	4.43	1.85	5.98		Unknown
16780133	6.18	3.64	5.84	SLITRK6	SLIT and NTRK-like family, member 6
17088446	6.71	4.18	5.8	LOC105376235	uncharacterized LOC105376235
17117867	7.71	5.34	5.18		Unknown
16734877	4.85	7.3	-5.46	HBE1	hemoglobin, epsilon 1
17001985	2.12	4.62	-5.68	LINC01470	long intergenic non-protein coding RNA 1470
17088527	3.76	6.35	-6.03	TLR4	toll-like receptor 4
16708796	3.3	6.03	-6.66	INA	internexin neuronal intermediate filament protein, alpha
17070482	3.77	6.67	-7.44		Unknown
16940203	3.69	6.62	-7.61	RTP3	receptor (chemosensory) transporter protein 3
16734902	3.81	6.78	-7.85	OR51I1;	olfactory receptor, family 51, subfamily I,
16865782	4.71	7.72	-8.05	RFPL4AL1	ret finger protein-like 4A-like 1
16921456	5.47	8.56	-8.52		Unknown
16853375	4.34	7.65	-9.91		Unknown
17024315	3.18	7.11	-15.35		Unknown
16734898	2.23	6.22	-15.88	OR51B2	olfactory receptor, family 51, subfamily B, member 2 (gene/pseudogene)
16734886	3.37	7.57	-18.31	HBG2; HBE1	hemoglobin, gamma G; hemoglobin, epsilon 1
17020497	3.01	7.37	-20.54		Unknown
16734883	3.13	7.9	-27.2	OR51B4	olfactory receptor, family 51, subfamily B, member 4

Gene chips (Human Gene 2.0 ST array, Affymetrix) were hybridized with 143B, Bkid, and Blym RNA each other. Total 48226 transcript clusters (tc) were analyzed, and the results were initially cut by the threshold 2.0 of fold increase. Bkid vs 143B showed unique 523 tc, and Blym vs 143B showed unique 273 tc. Thus, Bkid cells were most differentiated cell line. We further increased the threshold to 5, and cut off the genes in the comparison between Bkid vs 143B. The results were listed in the table.

Regarding cell morphology, Bkid formed a cohesive sheet compared to 143B (**Figure 2I**). Thus, we searched for cell-cell adhesion molecules in the microarray and found that R-, K-, and N-cadherin were upregulated in Bkid cells (**Figure 2J**). N-cadherin upregulation was unique to Bkid cells (**Figure 2K**). To choose the target of interest among the candidates, we examined them by overexpressed them in 143B cells (data not shown), and found that N-, OB-, and K-cadherin were increased when PEDF was overexpressed in 143B cells (**Figure 2L**). Therefore, we investigated the role of PEDF in Bkid cells.

### PEDF Knockdown in Bkid Cells Blocks Renal Metastasis

**Figure 3:**
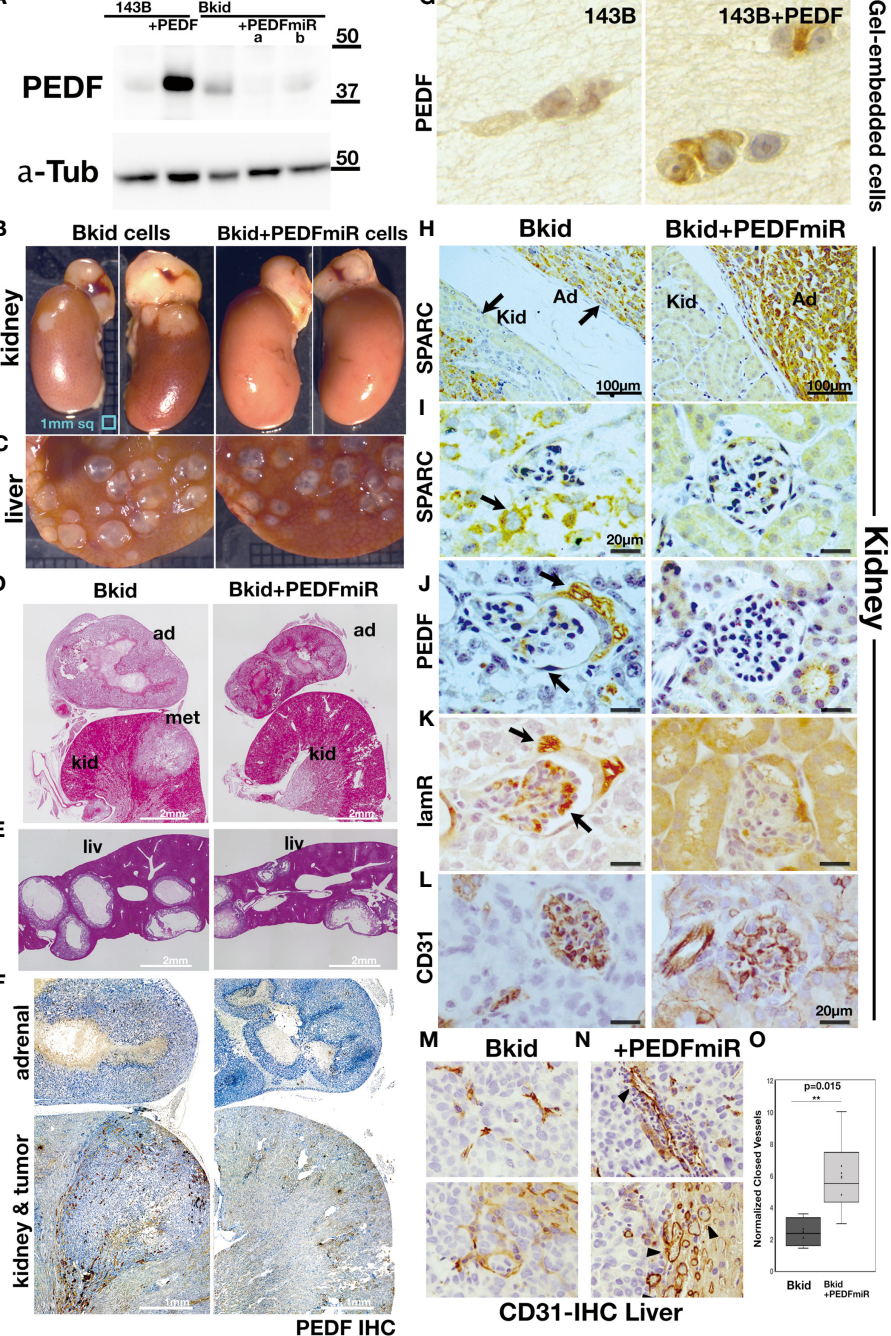
Loss-of-function analyses of PEDF in Bkid cells. **(A)** PEDF micro RNA (miR) expressing vector was transfected into Bkid cells. Immunoblot by anti-PEDF antibody showed the reduction of PEDF proteins by miRs. **(B)** The renal and the hepatic lesions of the results of the intracardiac injections of Bkid cells or Bkid+PEDFmiR cells. The adrenal metastasis was observed. Also, by Bkid cell injections, the renal metastasis was observed frequently (left, 12-positive/12), while no renal nodule was observed in Bkid+PEDFmiR injected mouse (right, 17-negative/18). The ruler is 1mm square. **(C)** The hepatic metastases of both cells were not inhibited with or without PEDF. **(D)** The section Hematoxylin/Eosin (H&E) staining of the similar samples to **(B)**. The adrenal hypertrophy was observed both in Bkid- and Bkid+PEDFmiR-injected mouse organs. The renal metastatic lesion (met) was seen as the lighter color. The scale is 2 mm. **(E)** The hepatic metastasis. Both tumors had the cavity. **(F)** PEDF-immunohistochemistry (IHC) of the section **(D)**. PEDF staining was observed inside of the adrenal gland cystic vesicle of Bkid tumors. The overall staining level of PEDF in Bkid tumor was higher than Bkid+PEDFmiR cells. The scale is 1 mm. **(G)** IHC for PEDF on paraffin sections of collagen gel-embedded 143B and 143B+PEDF cells. (H-L) The renal section IHC by various antibodies. **(H)** Anti-SPARC (osteonectin)-IHC indicates that the lesions are derived from the injected osteosarcoma. In the section of kidney from Bkid-injected mouse, there is SPARC-positive tumor both in kidney and adrenal gland sides. On the right, the kidney from Bkid+PEDFmiR-injected mouse had SPARC-positive cells only in adrenal gland. The scale indicates 100 µm. **(I–L)** The scale indicates 20 µm. **(I)** The higher magnified picture of the tissue surrounded glomerulus. There is SPARC-positive cells were seen around the glomerulus of Bkid-injected mouse (arrow), while no signal was seen in Bkid+PEDFmiR-injected mouse, **(J)** PEDF-IHC. PEDF was accumulated in the podocytes around the glomerulus. **(K)** 67kDa laminin receptor (lamR)-IHC. lamR is a potential PEDF receptor. Arrows indicated lamR signals in the podocytes around and the vein in the glomerulus. **(L)** CD31-IHC in glomerulus. **(M, N)** The liver section IHCs with CD31 antibody. The vascular formation was observed. **(M)** No closed blood vessel was seen in Bkid tumor (above). Near the tumor-liver boundary in Bkid sample (below). **(N)** The blood vessels could be seen as the closed oval form in Bkid+PEDFmiR tumor (above). Tumor-liver boundary of Bkid+PEDFmiR (below). There were a lot of the closed blood vessels (arrowheads). **(O)** The closed shapes of CD31-staining were counted only in the tumor regions, and the results were normalized by CD31-positive area. PEDF expression severely disturbed the formation of blood vessels (p=0.015). ad, adrenal gland; kid, kidney; met, metastatic lesion; liv, liver.

To determine whether PEDF is responsible for the renal metastasis of Bkid cells, we performed loss-of-function analyses. PEDF protein was mostly reduced by transfection with the microRNA (miR) expression vector (**Figure 3A**). Next, we examined the renal metastasis of Bkid cells with PEDFmiR, and confirmed that Bkid cells had metastasized into the kidney 20 days after their cardiac injection (**Figure 3B**, left), whereas PEDF-knockdown Bkid cells (Bkid+PEDFmiR cells) did not metastasize to the kidney (**Figure 3B**, right column). However, PEDF knockdown did not block the hepatic metastasis (**Figure 3C**). A tumor was found inside the kidney of Bkid-injected mice (**Figure 3D**, left, met), but not in Bkid+ PEDFmiR-injected mice (**Figure 3D**, right). In the liver, both cells formed nodules (**Figure 3E**), with no difference in the number or size between the hepatic lesions of Bkid and Bkid+PEDFmiR cells. First, the overall levels of PEDF were tested in the Bkid tumor compared to Bkid+PEDFmiR (**Figure 3F**). The specificity of PEDF-IHC was tested by staining the gel-embedded cells (**Figure 3G**). To determine whether the cells still have osteosarcoma properties, we examined SPARC/osteonectin expression (39) in the kidney (**Figures 3H, I**). SPARC expression was observed in both the kidney and adrenal glands of Bkid (**Figure 3H**, left, arrows), whereas only in adrenal gland of Bkid+PEDFmiR (**Figure 3H**, right).

### PEDF Ligands Are Found on the Cellular Surface of Kidney or Kidney Vein Cells

Next, we investigated the expression of PEDF-related genes (**Figures 3J, K**). PEDF expression was diffusible, which was mostly observed in the cavity of the adrenal gland lesions rather than in the cancer cells (**Figure 3F**). Close observation revealed that PEDF expression was localized on the surface of podocytes around the glomerulus and inside the glomerulus (**Figure 3J**). We presumed that the PEDF ligand might be anchored to the cell surface receptor. In a similar section, we found that one of the PEDF receptor molecules, laminin receptor/67lr (lamR) (14), accumulated on podocytes and glomeruli (**Figure 3K**); however, PEDF and lamR were faint or negative in the kidneys of Bkid+PEDFmiR-injected mice (**Figures 3J, K**, right columns). Therefore, PEDF proteins may be secreted from infiltrated cancer cells and attached to kidney podocytes or kidney vascular cells (**Figure 3L**).

### PEDF Reduced Blood Vessels in Liver Tumors

Both Bkid and Bkid+PEDFmiR cells were able to infiltrate the liver (**Figures 3C, E**), possibly because the acquisition of PEDF in Bkid occurred after differentiation from Bliv cells (**Figure 2H**). PEDF is known to inhibit angiogenesis or tumor neovascularization (13, 20). We were not able to compare this function in the kidney because only Bkid had renal metastasis; thus, we performed IHC of CD31 in liver tissues (**Figures 3M, N**) (40). In Bkid-injected mice, CD31-positive cells did not shape the closed blood vessels (**Figure 3M**, above), while the closed vessels were observed in Bkid+PEDFmiR tumors (**Figure 3N**, above), and the tumor-liver tissue border showed it more clearly (**Figures 3M, N**, below). The number of closed vessels was significantly lower in the Bkid tumors (**Figure 3O**).

Therefore, these data suggest that either Bkid or Bkid lacking PEDF could form hepatic metastatic lesions in the 143B nature; however, the function of PEDF as a neovascularization inhibitor was intact in the liver tumor. Moreover, hepatic phenotypes were not involved in the PEDF acquisition in Bkid.

### 143B+PEDF Quickly Causes Pulmonary Metastasis

**Figure 4:**
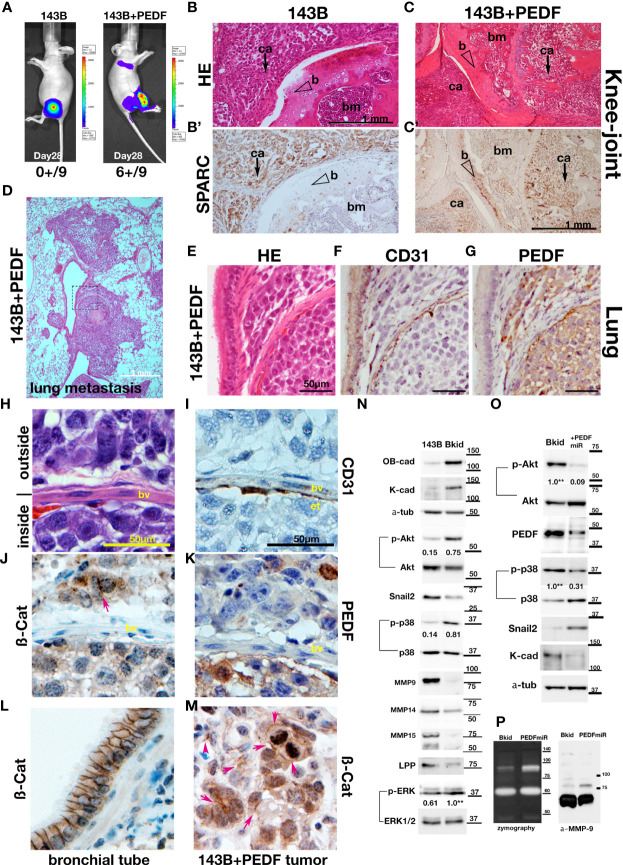
PEDF overexpression promoted the MET-like behavior in pulmonary metastasis. **(A–K, M)** The results of higher doses (2 x 10^6^ cells) injections into the knee joints. **(A)** *In vivo* luciferase-imaging of 143B/143B+PEDF cells after 28 days from the injection. The signals were observed in the lung of 143B+PEDF cell-injected mice (right, six positive/10: one dead), while no signal was seen in thorasic region of 143B-injected mice (left, zero positive/nine). **(B, C)** The H&E stain of knee joint and femur section. The scale bar indicates 1mm. **(B’, C’)** SPARC-IHC of the adjacent section of **(B, C)**. The scale bar indicates 1 mm. **(B)** The 143B cells (ca; arrow) were mainly observed outside of the knee bone. **(B’)** SPARC-IHC. The cells outside of the joint were osteosarcomas. **(C)** 143B+PEDF cells were observed inside of the bone (ca; arrow). **(C’)** SPARC-IHC of **(C)**. The SPARC-positive cancer cells were seen both in the bone marrow (bm) and the joint. The opened arrowheads mean the position of the bone. **(D)** The H&E stain of the pulmonary metastatic lesion of the 143B+PEDF-injected mouse. The square of the broken line is the region of interest in **(E–G)**. **(E–G)** The lung vascular border in 143B+PEDF cell-injected mouse at higher magnification. The cancer cells inside and outside the vasculature are connected with each other. The scale bar indicates 50 µm. **(E)** shows H&E staining of these sections. **(F)** shows IHC for CD31 on a similar section. The endothelial cell layer is very thin. **(G)** shows IHC for PEDF. PEDF-positive cells are present both inside and outside the vein. (H–K, M) The 143B+PEDF tumor in the lung at higher magnification. The scale bar indicates 50 µm. **(H)** shows H&E staining of the inside and outside of vasculature. **(I)** shows IHC for CD31 indicating the endothelial layer. **(J)** shows IHC for ß-catenin on both sides of the boundary. **(K)** shows IHC for PEDF also on both the sides. **(L)** IHC for ß-catenin-in the normal bronchial tube near the tumor lesion. **(M)** IHC for ß-catenin in the tumor far away from the vascular boundary. Arrowheads indicate strong staining on cell–cell junctions. **(N)** Immunoblot of 143B and Bkid cell lysates. OB-cadherin and K-cadherin was upregulated, whereas Snail2 was downregulated. The ratios of phosphorylated to whole Akt for each sample are shown between the blots. The ratio of phosphorylated to whole p38MAPK, and the ratio of phosphorylated to whole ERK1/2 are also shown between each blot. Expression of MMP9 was diminished in Bkid cells, whereas that of MMP14, MMP15, and LPP was decreased. **(O)** Comparisons between Bkid and Bkid+PEDFmiR cell lysates. The amount of PEDF were reduced. The ratios of phosphorylated to whole Akt and phospho-p38MAPK to whole p38 are also shown between the blots. The phosphorylation level decreased upon PEDF knockdown. Snail2 was upregulated in Bkid+PEDFmiR, whereas K-cadherin was downregulated. **(P)** Zymography indicating that metalloprotease activity was increased upon PEDF knockdown. The upregulated MMP was mainly MMP9.

The most common destination for osteosarcoma metastasis is the lung; thus, to test the lung infiltration ability of 143B or 143B +PEDF, we performed knee-joint injections of these cell lines and monitored them using *in vivo* imaging (**Figure 4A**). The 143B +PEDF cell line metastasized to the lung within 28 days (**Figure 4A**, right; six positive out of nine mice, one died earlier), whereas 143B parental cell lines did not show any metastasis during the same period (**Figure 4A**, left; zero positive in thorasic level out of nine mice). The 143B cells were observed around the knee joint (**Figure 4B**), whereas 143B +PEDF cells efficiently invaded the bone marrow of the femur (**Figure 4C**), which was confirmed by SPARC staining (**Figures 4B, C**). We also analyzed the pulmonary metastatic lesions (**Figures 4D–G**) and found that 143B cells were not seen in the lungs (data not shown), whereas the 143B +PEDF cells infiltrated the lung tissues or blood vessels (**Figure 4D**). The vascular wall (endothelial cells) had already been thin in the metastatic lesion (**Figures 4E, F**); further, PEDF staining was observed in these tumors (**Figure 4G**). We closely observed the cell-cell adhesions inside and outside the blood vessels (**Figures 4H–K, M**). The endothelial layer was shown by CD31 staining (**Figure 4I**), and both β-catenin and PEDF signals were observed on both sides of the vascular boundary (**Figures 4J, K**), and ß-catenin IHC was accumulated in the cell-cell junctions (**Figure 4J**, arrow). When we looked at the position far away from the blood vessels, many 143B +PEDF cells formed cell-cell adhesions in the lung (**Figure 4M**), while the bronchial tube showed similar staining (**Figure 4L**). Therefore, these data suggest that PEDF may promote mesenchymal-to-epithelial transition (MET) after extravasation.

### PEDF Promotes Mesenchymal-to-Epithelial Transition (MET)

To understand why PEDF increased metastatic lesions, we analyzed the protein expression of 143B/Bkid. As mentioned above, K- and OB-cadherins were upregulated by PEDF (**Figure 4N**). Phosphorylation of Akt and p38MAPK signaling was also upregulated in Bkid cells (**Figure 4N**). We found that epithelial-to-mesenchymal transition (EMT)-related markers, such as Snail2, MMP9, and MMP14, were downregulated in Bkid cells (**Figure 4N**). Moreover, we previously reported that MMP15, under the control of the transcriptional co-factor LPP, digests N-cadherin in lung cancer cells, and that loss of LPP function caused abnormal stabilization of N-cadherin (27). Similarly, in Bkid cells, MMP15 and LPP were downregulated (**Figure 4N**). Thus, these results indicate that Bkid partially promotes MET. To determine if this MET phenotype is due to PEDF expression, we compared Bkid cells and PEDF-knockdown cells (**Figure 4O**). PEDF-knockdown in Bkid cells resulted in a reduction in the phosphorylation of Akt and p38MAPK, and upregulation of Snail2 (**Figure 4O**). Moreover, K-cadherin expression was reduced (**Figure 4O**). Further, zymography of Bkid and PEDFmiR cell supernatants showed that the activity of MMP-9 was also upregulated in PEDF-knockdown cells (**Figure 4P**). Therefore, PEDF expression in Bkid cells promoted MET phenotypes, at least partially.

### PEDF Delays Cell Delamination Without Affecting Cell Proliferation

**Figure 5:**
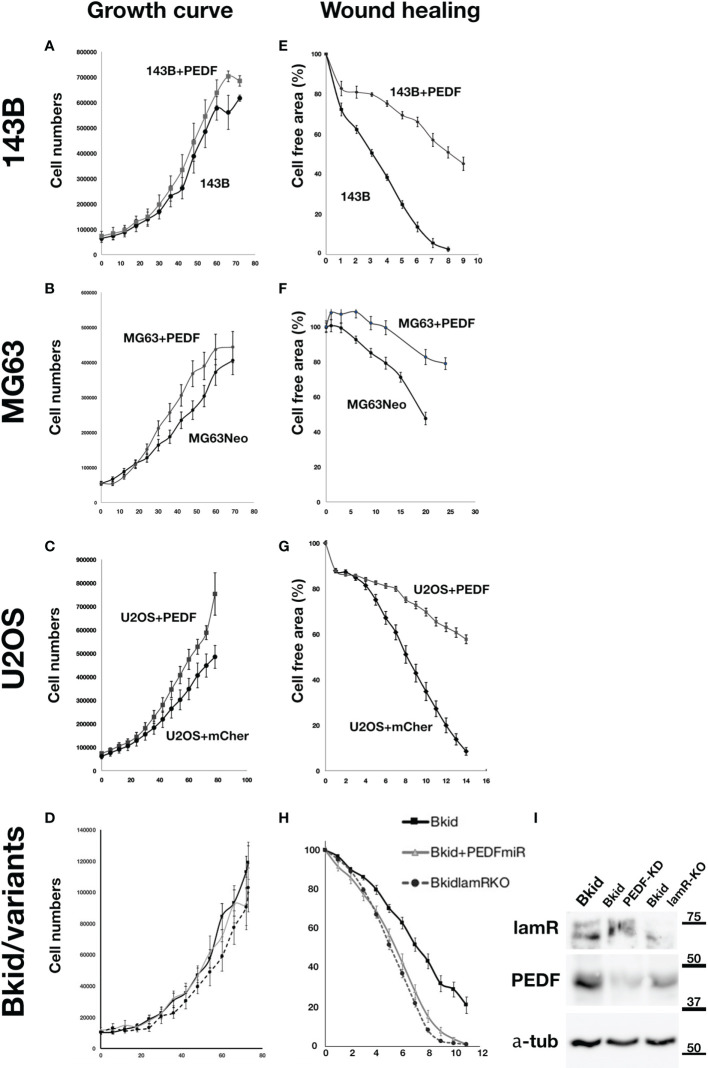
PEDF did not inhibit the proliferation but inhibited the migration. **(A–D)** Cell numbers over time. **(E–H)** The decrease ratio of the cell free area by wound healing. **(A)** The growth curves of 143B and 143B+PEDF cells. PEDF overexpression slightly increased the cell proliferation, but the differences were not significant. **(B)** The cell numbers of MG63+MOC vector (MG63Neo) and MG63+PEDF in cell culture. MG63+PEDF grow slightly faster than MG63Neo. **(C)** The cell numbers of U2OS+mCherry vector and U2OS+PEDF in cell culture. Likely U2OS+PEDF grow faster than that of U2OS+mCherry. **(D)** The comparisons among Bkid, Bkid+PEDFmiR, and Bkid lamR knockout cells. The proliferations were not significantly changed by loss-of-function. **(E)** Wound healing abilities of 143B and 143B+PEDF cells. PEDF overexpression decreased cell motility of 143B. **(F)** Wound healing of MG63Neo and MG63+PEDF. MG63+PEDF delayed the healing. **(G)** Wound healing of U2OS+mCherry and U2OS+PEDF. Same as other cells, U2OS+PEDF significantly delayed the healing. **(H)** The wound healing phenotype of Bkid was cancelled by the knockdown of PEDF-lamR pathway. **(I)** Western blot of the effect of gene knockdown and gene knockout cell lysates. LamR knockout and PEDF knock down did not affect on the other’s expression each other.

The experiments above showed that PEDF caused an MET-promoting phenotype; thus, we examined the MET marker expression to determine the general role in the osteosarcomas; however, the basal expressions of osteosarcomas were very different from each other (data not shown). Therefore, we examined whether PEDF overexpression had similar effects on other osteosarcomas. First, the effect of PEDF on cell proliferation was tested in 143B, MG63, and U2OS cells (**Figures 5A–C**). The proliferation of PEDF-overexpressing cells was slightly higher than that of the control cells (**Figure 5A-C**). Furthermore, we examined the autocrine effects of PEDF in Bkid cells (**Figure 5D**). Neither PEDF knockdown nor laminin receptor knockout affected cell proliferation (**Figure 5D**). We also examined the wound healing abilities of each cell line because PEDF might block cell delamination by increasing the epithelial-like character (**Figures 5E–H**). PEDF significantly delayed wound healing in MG63 and U2OS cells, similar to that in 143B cells (**Figures 5E–G**). Loss-of-PEDF or lamR showed faster healing (**Figure 5H**), while only the targeted protein was reduced (**Figure 5I**). Therefore, PEDF expression caused MET-promoting phenotypes in other osteosarcoma cells without changing cell proliferation.

### Low Numbers of 143B+PEDF Cells Infiltrate Into the Liver and Kidney

As mentioned above, the orthotopic injection of PEDF-overexpressing 143B caused pulmonary metastasis (**Figure 4**), and the mice died of respiratory failure before showing renal metastasis. Thus, we injected smaller amounts of cancer cells and observed them for a much longer period (**Supplementary Figure 2**). The leg tumors of 143B +PEDF were larger than those of 143B (**Supplementary Figures 2A, B**). While the hepatic metastasis was not obvious because the superficial nodules did not appear (**Supplementary Figure 2C**), and the renal phenotype was clearly observed (**Supplementary Figure 2D**). In the liver sections, the inflammatory cells accumulated around the central vein; however, no apparent metastatic tumor was observed (**Supplementary Figures 2E–H**). On the other hand, a renal metastatic tumor was observed (**Supplementary Figures 2I, J**), and the cancer cells surrounded the glomerulus in 143B +PEDF cell-injected mice (**Supplementary Figures 2K–N**). Therefore, PEDF overexpression in 143B could also bias metastasis toward the kidney.

### PEDF-R and Laminin Receptor Expression in the Kidney, Liver, and Lung

**Figure 6:**
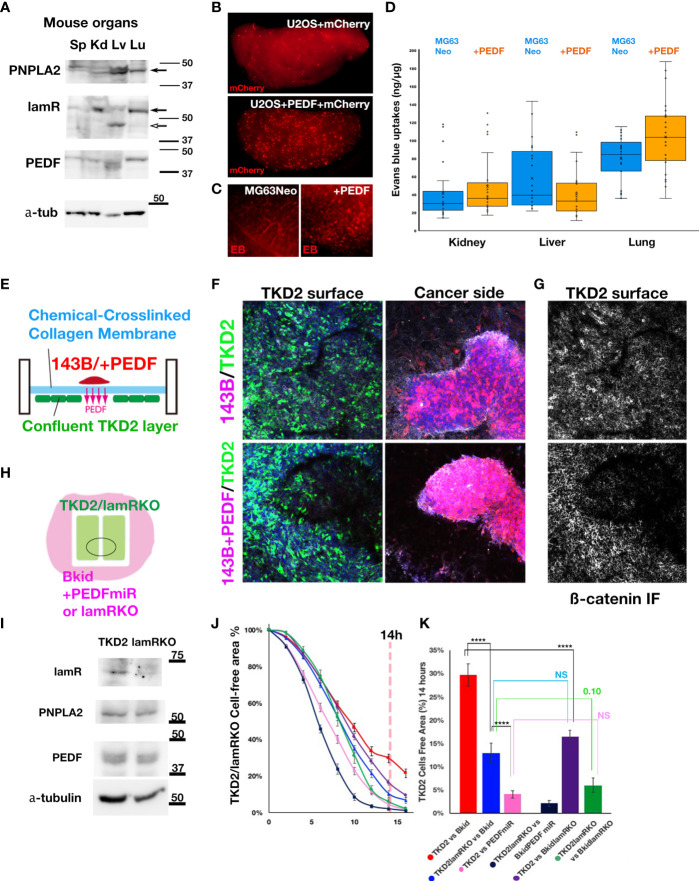
The effects of PEDF on extravasation and inhibition of EndoMT. **(A)** Immunoblot of mouse spleen (Sp), kidney (Kd), liver (Lv), and lung (Lu) lysates by anti-PEDF receptor molecules. PNPLA2/PEDF-R was expressed in liver and lung. Laminin receptor/67lr (lamR) was expressed in the kidney and the lung (arrow). The lamR in liver sample was truncated (open arrow). The endogenous PEDF was not significantly different among these organs. **(B)** The cells extravasated in the lung of tail intravenous injected U2OS+mCherry (above). The cells extravasated in the lung of U2OS+PEDF (+mCherry) injected mouse. The mCherry-positive cells were observed everywhere in the lung (below). **(C)** After two weeks of the intracardiac injection of MG63Neo or MG63+PEDF, Evans Blue (EB) dye was injected from the tail vein two hours before the sacrifice. EB dye was visualized as the red fluorescence, and when the vascular permeability was increased, the EB dye remained in the organs (See *Materials & Methods*). In MG63+PEDF-injected mouse lung, the large puncta of EB remained (right) while the small vein was stained in control (left). **(D)** The quantitative analyses of Milles assay shown in **(C)**. The remained EB dyes were extracted from each dissected organ by the heated formamide and quantified by OD_600_. **(E)** The schematic illustration of the double-sided cell culture on the atelocollagen membrane. The mouse kidney endothelial cell line, TKD2 cells expressing GFP (TKD2-GFP) were cultured on the one side of the chemically cross-linked atelocollagen membrane, and the appropriate size of 143B cell cluster was transferred on the other side of the membrane. We could see the localized effects of PEDF because only secretory molecules could go across this cross-linked membrane (See *Material and Methods*). **(F, G)** The Z-stack images were taken under the confocal microscopy. The nucleus of the cells (blue), TKD2-GFP (green), osteosarcomas (red), and ß-catenin immunofluorescence (IF) (white) images were merged. **(F)** TKD2 layer was disturbed by 143B+PEDF. The optical section of TKD2 side (above). The projection image of cancer side (below). **(G)** ß-catenin IF of TKD2 layer. ß-catenin staining was disappeared from the region where the opposite side were occupied by the cancer cells. **(H)** The schematic diagram of the wound healing assay of TKD2-derived cell layers. TKD2 cell layers were formed inside of the silicon molds. The cancer cells were cultured around the mold, and time-lapse images were captured after removing the mold. **(I)** Western blot analyses of TKD2-laminin receptor knockout cells (lamRKO). The PEDF or its receptor expressions were not changed. **(J, K)** [TKD2 migrations with Bkid (red)], [TKD2 with Bkid+PEDFmiR (pink)], [TKD2-lamRKO cell migrations with Bkid (blue)] and [TKD2-lamRKO with Bkid+PEDFmiR (dark blue)], [TKD2 with Bkid-lamRKO(purple)] and [lamRKO-TKD2 with Bkid-lamRKO (green)]. **(K)** The graph indicated the cell free area at 14 hours after the start. PEDF knockdown in Bkid cells or lamR knockout in TKD2 accelerated the endothelial cell migration. NS, Not significant. The symbol **** means 4 digit below the decimal point, enough significant difference according to the result of t-test.

To understand why Bkid cells prefer to infiltrate the kidney or lung, an understanding of endogenous receptor expression in mouse organs is necessary. The phospholipase molecule PNPLA2 is a putative PEDF receptor (41). The other receptor molecule laminin receptor/67lr (lamR) (14), has already been described above. Thus, we performed western blot analyses of the endogenous protein levels of these genes in mouse organs (**Figure 6A**). PNPLA2 was strongly expressed in the liver and weakly expressed in the lungs and kidneys (**Figure 6A**, arrow). LamR was clearly expressed in the kidneys and lungs (**Figure 6A**, arrow), whereas it was truncated in the liver (**Figure 6A**, open arrow). Thus, the phenotype of Bkid in the kidney may be mediated by the laminin receptor.

### PEDF Overexpression Induces Extravasation of Osteosarcoma Cell Lines

We hypothesized that MET phenotypes caused by PEDF overexpression might obstruct extravasation. Then, we performed tail vein injection of U2OS+mCherry cells with or without PEDF, and fixed the lung two hour after injection (**Figure 6B**). Contrary to our initial expectation, PEDF increased the extravasation of cells in the lung (**Figure 6B**). To confirm these results, we injected MG63+Neo (MOC vector) cells or MG63+PEDF cells in the heart, and after two weeks, we injected Evans Blue (EB) dye from the tail vein, and then dissected the lung (**Figure 6C**). Because the molecular weight of EB dye is almost the same as the membrane pore size, the Milles assay can be used to indicate increased vascular permeability (31). MG63 could not survive for more than two weeks (data not shown), suggesting that PEDF overexpression increased vascular permeability (**Figure 6C**, right). We further quantified the remaining EB in each organ (**Figure 6D**). The estimated amounts of EB seemed to be increased in the kidneys and lungs by PEDF overexpression (**Figure 6D**), while the liver did not show such an increase. These data suggest that PEDF overexpression in osteosarcoma may increase vascular permeability in the kidneys and lungs.

### PEDF Inhibits Endothelial Cell Layer Formation *via* Laminin Receptor

To determine how PEDF affects extravasation, we focused on the layer-forming ability of TKD2, a kidney endothelial cell line. TKD2 could be a model for the glomerular endothelial cells (42), which may be suitable for testing the extravasation. Thus, we designed the assay to observe the interaction between the endothelial layer and the cancer cells (**Figure 6E**). A confluent TKD2 cell layer was prepared on the bottom side of the culture insert, and a cancer cluster was prepared separately, which was then placed on the atelocollagen membrane. The membrane is chemically cross-linked; thus, the cells never across the membrane. However, the effect of the secretory molecules can be distinctly observed (**Figure 6E**). The cluster of 143B+PEDF reduced the number of TKD2 cells on the opposite side of the cancer cluster (**Figure 6F**, below), whereas the cluster of 143B did not disturb theTKD2 layer (**Figure 6F**, above). The ß-catenin immuno fluorescence staining showed the cell-cell adhesions of TKD2 were highly disturbed by PEDF overexpression (**Figure 6G**, below). We conducted similar assay with Bkid and Bkid+PEDFmiR cells (**Supplementary Figure 3A, B**), and observed that Bkid+PEDFmiR cells did not disturb the TKD2 layer (**Supplementary Figure 3B**). Therefore, we inferred that the close contact between PEDF-overexpressing and endothelial cells might have increased cell permeability. Next, to analyze the cell-cell interactions more quantitatively, TKD2 and 143B cells (or derivative cells) were separated using a silicon mold, and the cell-free area between TKD2 layers was then observed (**Figure 6H**). TKD2 migrated rapidly when co-cultured with 143B cells, whereas PEDF overexpression delayed their migration over time (**Supplementary Figure 3C**). We established a TKD2-lamR knockout cell line without changing the expression of PNPLA2 or PEDF (**Figure 6I**). Next, we tested various combinations of TKD2 with or without lamR, and Bkid with or without PEDF (**Figure 6J**). When lamR expression in TKD2 cells and PEDF expression in Bkid cells was inhibited, the endothelial cells migrated at the highest speed (**Figure 6J**, dark blue). The results at 14 h were compared (**Figure 6K**, pANOVA=3.37E-15), and the t-test of each pair was also performed (**Figure 6K**, ****p<0.001). These data suggest that PEDF expression in Bkid cells effectively inhibited the layer formation process in renal endothelial cells, and the signals for this inhibitory process might be mediated by lamR in TKD2 cells.

### PEDF-lamR Downstream in TKD2-Endothelial Cells

**Figure 7:**
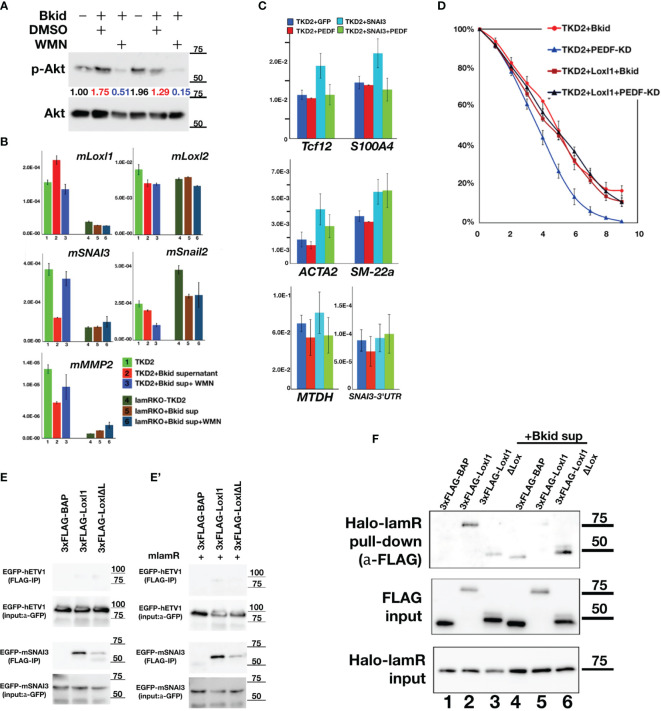
PEDF-lamR downstream in TKD2 endothelial cells. **(A)** The phosphorylation of Akt in TKD2 cells were stimulated by Bkid supernatant and inhibited by wortmanin (WMN) treatment. The numbers showed the ratio of phosphorylated Akt to Akt. **(B)** The qPCR of the same sample as **(A)**. *Lysyl-oxidase like 1*(*Loxl1*) was increased by Bkid stimulation and blocked by WMN. Conversely, *SNAI3* (*Snail3*) expression was downregulated by Bkid and increased by Bkid+WMN, while *Loxl2* and *Snail2* were not affected by such stimulations. *MMP2* were regulated similarly to *SNAI3*. **(C)** The gene expressions of PEDF-overexpressing, SNAI3-overexpressing and SNAI3+PEDF-coexpressing TKD2. Endothelial-to-mesenchymal Transition (EndoMT) markers were tested. *Tcf12*, *S100A4* (fibroblastic), *ACTA2* (fibroblastic) markers indicated that SNAI3 overexpression promoted the EndoMT, and SNAI3 function was blocked by PEDF-coexpression. *SM-22a* (smooth muscle protein) was not inhibited by PEDF. The known EMT marker, *Metadherin* (*MTDH*) was also upregulated by SNAI3 overexpression. The endogenous *SNAI3* was not significantly changed by the overexpression. **(D)** TKD2+Loxl1 cell line was established. The layer formation of TKD2 with Bkid+PEDF-KD was promoted, however, TKD2+Loxl1 with Bkid+PEDF-KD was inhibited as same as that of TKD2+Bkid. **(E, E’)** The co-immunoprecipitation between 3xFLAG-Loxl1 and EGFP-ETV1 (negative control of EGFP-tag) and EGFP-SNAI3. FLAG-tagged bovine alkaline phosphatase (3xFLAG-BAP) was negative control of FLAG-tag. Loxl1 pulled down SNAI3 while Loxl1 lacking Lox domain (Loxl1ΔL) could not bind to SNAI3 same way as full-length Loxl1, thus, the center of binding between SNAI3 and Loxl1 is the Lox domain of Loxl1. **(E’)** The co-expression of lamR protein did not affect on the binding between Loxl1 and SNAI3. **(F)** The binding between HaloTag-fusion protein of laminin receptor (Halo-lamR) and 3xFLAG-Loxl1 was tested with or without Bkid stimulations. Loxl1 could bind to lamR (lane 2), while Bkid stimulation released Loxl1 from Halo-lamR (lane 5). Non-specific binding was slightly increased after adding Bkid proteins (lane 4).

The PEDF-lamR axis is known to be mediated by Akt phosphorylation (43). First, we analyzed the expression of Snail2 or the other MET-related markers by western blotting, as shown in **Figure 4**; however, mouse Snail2 was not detected by this method (data not shown). Therefore, we investigated the genes targeted by lamR using the Akt inhibitor wortmannin (WMN) (**Figure 7A**). The condensed Bkid supernatant (Bkid sup) increased Akt phosphorylation, and the basal level of phosphorylation was high because TKD2 culture medium supplemented with epidermal growth factor. WMN treatment reduced Akt phosphorylation (**Figure 7A**), and the expression of various genes involved in this process was analyzed (**Figure 7B**). *Lysyl oxidase-like 1* (*Loxl1*) expression was upregulated by Bkid stimulation and inhibited by the addition of WMN. We analyzed the expressions of Snail1 and 2, however, no significant change in their expression was observed (**Figure 7B**, data not shown). We found that *SNAI3* expression was inhibited by the Bkid supernatant and upregulated by the addition of WMN. Thus, we identified Loxl1 and SNAI3 as candidate LamR downstream genes. SNAI3 overexpression in TKD2 cells increased the expression of marker associated with endothelial-to-mesenchymal transition (EndoMT), and co-expression SNAI3 with PEDF cancelled the EndoMT-upregulation by SNAI3 (**Figure 7C**). *Tcf12* is an osteopontin upstream gene that is involved in EndoMT (44). *S100A4* and *ACTA2* are both fibroblastic markers (45, 46), and their upregulation may indicate transient EndoMT. *SM-22a*, also known as *transgelin*, is a smooth muscle cell protein, which serves as an EndoMT marker (47). *SM-22a* upregulation by SNAI3 overexpression was not inhibited by co-expression of PEDF; thus, we inferred that strict regulation of SNAI3 expression was required for the maintenance of the endothelial cell layer. We also found that the known EMT marker, metadherin (48), was expressed with the occurrence of EndoMT. Endogenous *SNAI3* levels in SNAI3 overexpressing TKD2 cells was monitored; however, no significant difference was observed in *SNAI3* levels (**Figure 7C**). We established Loxl1-overexpressing TKD2 and conducted the same assay, as shown in **Figure 6E**. TKD2-Loxl1 blocked the effects of PEDF-knockdown Bkid cells (**Figure 7D**). We infer that SNAI3 promotes EndoMT, and its expression is enhanced by Akt inhibition. In contrast, Loxl1 inhibits EndoMT, and its expression is promoted by Bkid cell stimulation. Next, we tested whether SNAI3 and Loxl1 directly interacted with each other; For this purpose, we first examined the binding affinity between Loxl1 and SNAI3 (**Figure 7E**). SNAI3 mainly bound to the Lox domain of Loxl1 (**Figure 7E**), and lamR expression did not disrupt the binding affinity between SNAI3 and Loxl1 (**Figure 7E’**). Next, we then tested the binding affinity between lamR and Loxl1 (**Figure 7F**). In absence of Bkid sup stimulation, Loxl1 bound to lamR (**Figure 7F**, lane 2). However, the binding affinity was disrupted under the Bkid sup stimulation (**Figure 7F**, lane 5). In summary, Loxl1 may associate with lamR until Bkid cell stimulation, and the released Loxl1 may bind to SNAI3 and inhibit EndoMT upon endothelial layer formation.

Collectively, our results suggest that PEDF expression increases the permeability of kidney and lung vasculature owing to its association with endogenous lamR expression. Thus, the PEDF-lamR axis may promote the extravasation of cancer cells and may be involved in target organ specificity.

## Discussion

We sought to identify the mechanisms underlying the organ tropism of metastasis in osteosarcoma; however, the candidate for the renal metastasis-specific gene was unexpectedly an anti-cancer molecule, PEDF. Until now, PEDF function has been examined in a wide range of cancers, including osteosarcoma, prostate cancer, breast cancer, and hepatocellular carcinoma (19, 20, 43, 49–51). PEDF shows anti-angiogenic, anti-tumor, and anti-metastatic effects (18, 19, 22, 23, 52). However, the role of endogenous PEDFs in cancer cells has not been thoroughly investigated. Here, we discuss the differences between previous studies and the present study. Interestingly, we found that PEDF only affected renal and pulmonary metastases; therefore, we could conclude that the PEDF-lamR axis also controlled organ tropism.

PEDF is a promising candidate for anticancer drugs. However, we observed contrasting effects of PEDF in osteosarcoma cell lines, possibly because of the following reasons. First, the anti-angiogenic activity of PEDF was shown to inhibit tumor vascular formation (49, 53). Similar to previous observations, the inhibitory effect of PEDF on tumor vasculature was observed (**Figure 3O**); however, it did not interfere with hepatic metastasis (**Figure 3C**). This study suggests that organ vascular inhibition by PEDF may promote extravasation into the target organ (**Figures 6B–D**). At least some part, the phenotype of the extravasation was reproduced by the co-culture assay shown in **Figures 6E–G**.

The anti-tumorigenic activity of PEDF in OS has been previously reported in SaOS-2 (18). The synthetic peptide of PEDF has been demonstrated to exert anti-tumorigenic activity (52, 54). In addition, the administration of recombinant PEDF protein showed the therapeutic effects on the primary OS and the secondary pulmonary tumor in an orthotopic animal model using SaOS-2 (22). However, the researchers also reported that there were no significant differences in the mean number of pulmonary micrometastasis between treatment and non-treatment groups (22), and the similar observation from the synthetic peptides test was also reported (52). It was speculated that the proliferation in the secondary lesions may be involved in these phenotypes (22). In our study, PEDF overexpression in 143B increased the pulmonary metastatic lesions (**Figure 4D**) and MET (**Figure 4M**), which may be favorable for the local growth of metastasized cells in the lung. Moreover, PEDF also exerted anti-oxidative stress activity (16, 55). In fact, the cell survival signal was increased in Bkid cells but reduced following PEDF knockdown, as evidenced by the increase in the phosphorylation of Akt and p38MAPK (**Figures 4N, O**). Cell proliferation was slightly increased in three different cell lines (**Figure 5**), whereas PEDF inhibited proliferation and induced apoptosis in SaOS-2 (19). Additionally, PEDF causes necrosis in prostate cancer (49). Although Fas or death receptors are frequently negative in the pulmonary metastatic lesions of OS (1), cell death is still involved in the development of the secondary lesions. Therefore, the effects of endogenous PEDF on micrometastasis, cell survival, cell death, and the cell type-dependent differences should be carefully analyzed in future studies.

Third, PEDF has shown anti-metastatic effects in epithelial cancer cells (14, 19, 20). Notably, we showed that Bkid cells showed a decrease in EMT factors, which were reversed by PEDF knockdown (**Figures 4N, O**). It was reported that PEDF downregulated MMP9 and inhibit invasion in malignant U251 glioma (53), MMP downregulation is same as our result, but the output is opposite. Our study was performed in osteosarcoma, which is mesenchymal, and we skipped the early phase of metastasis, such as EMT and intravasation, by intracardiac injection (**Figures 1**, **3**). If PEDF blocked EMT in epithelial cancer cells in the early phase of metastasis, we also concluded that PEDF had anti-metastatic function; however, the same function could be used for MET after extravasation (**Figure 4**).

We also found novel molecular interactions among PEDF-lamR-Loxl1-SNAI3 (**Figure 7**). In Bkid cells, gain-of-function and loss-of-function analyses of PEDF showed the regulation of Snail2 (**Figures 4N, O**). We examined gene expression in TKD2, a renal endothelial cell line, but Snail2 expression was not detected in TKD2 using the same antibody used in **Figure 4N** (data not shown). We found the RNA expression of mouse Snail2 later in the qRT-PCR (**Figure 7B**); however, it still did not respond to PEDF stimulation (**Figure 7B**, data not shown). Thus, we once examined Snail1; however, it did not work as well (data not shown). Finally, we found that SNAI3 responded to Bkid stimulation and WMN treatment (**Figures 7A, B**). It has been reported that lysyl oxidase-like 2 (Loxl2) interacts with Snail (56). Again, Loxl2 did not respond to PEDF stimulation (**Figure 7B**). We examined Loxl1-4 expression (data not shown), and found that Loxl1 responds to stimulation (**Figure 7B**). The laminin receptor can bind to laminin and PEDF in the extracellular space, and it is also known as ribosomal protein SA (Rpsa) (15), which works at the inner cellular. Loxl proteins are extracellular enzymes involved in matrix modification (24), which work upon EMT (57–59); however, their cellular function has also been reported. Therefore, both Loxls and lamR have multi-distribution properties. We found that lamR could bind to Loxl1, and Bkid stimulation released Loxl1 freely. Because lamR did not interfere with the binding between Loxl1 and SNAI3 (**Figure 7E**), free Loxl1 may bind to SNAI3. We also found that Loxl1 overexpression was like PEDF overexpression in terms of inhibition of TKD2 migration (**Figures 6J**, **7D**), and that Loxl1 is promoted by Bkid stimulation under the control of lamR/Akt signaling (**Figures 7A, B**). Conversely, SNAI3 gene regulation was opposite to that in Loxl1, while SNAI3 enhanced EndoMT gene expression (**Figure 7C**). Therefore, it is reasonable to conclude that Loxl1 binds to SNAI3 to block transcription, and subsequently blocks EndoMT. Recently, the extracellular roles of lysyl oxidase (LOX) and these Loxl proteins have been reported (24, 58–61). LOX antibody-conjugated liposome was used for cancer-targeting drug deliverly because cancer cells secrete LOX during EMT (60). Further study on the inner cellular and extracellular roles and the potential feedback regulation of Loxl1 *via* PEDF is encouraged. Interestingly, pseudoexfoliation syndrome was associated with Loxl1 gene function (62), and it has been recently shown that PEDF expression level was also correlated with pseudoexfoliation (63). So far, there is no direct relationship between these studies, however, our finding may give them some connection.

Further study on patient specimens is a viable direction for future research. Our data suggested that the signal flow from PEDF to SNAI3 and its downstream molecules may control the extravasation and reorganization of tumor in specific target organs. In addition, the various combinations of these proteins may be key to predicting organ tropism of metastasis.

## Data Availability Statement

The datasets presented in this study can be found in online repositories. The names of the repository/repositories and accession number(s) can be found below: https://www.ncbi.nlm.nih.gov/geo/query/acc.cgi?acc=GSE188984.

## Ethics Statement

The animal study was reviewed and approved by Akita University Ethical Committee for Experimental Animals.

## Author Contributions

SK contributed to conception, design of this study, wrote the first draft of this manuscript, and edited the latest version of manuscript. GT, KT, and GI did data curation. MT read and edited the draft. All authors approved the submitted edition.

## Funding

SK was supported by JSPS KAKENHI (15K06822, 17KT0104, 18K07192, 21K07090) and the Takeda Science Foundation. MT was supported by JSPS KAKENHI (19H03495) and research grant from Princess Takamatsu Cancer Research Fund (19–25123).

## Conflict of Interest

The authors declare that the research was conducted in the absence of any commercial or financial relationships that could be construed as a potential conflict of interest.

## Publisher’s Note

All claims expressed in this article are solely those of the authors and do not necessarily represent those of their affiliated organizations, or those of the publisher, the editors and the reviewers. Any product that may be evaluated in this article, or claim that may be made by its manufacturer, is not guaranteed or endorsed by the publisher.
