# Supplementary material for: Pigment Epithelium Derived Factor Is Involved in the Late Phase of Osteosarcoma Metastasis by Increasing Extravasation and Cell-Cell Adhesion
Source: Front Oncol. 2022 Jan 31;12:818182. doi: 10.3389/fonc.2022.818182 (PMC8842676; doi:10.3389/fonc.2022.818182)
Supplement: Supplementary file 1 [file DataSheet_1.docx]

Supplementary Material

# Supplementary Data

**Supplemental Methods**

**Lentivirus and retrovirus vectors**

The lentivirus or the retrovirus vectors were transfected together with pCMV-VSV-G and pCMV-HIV-gag/pol (lentivirus) or pCL-10A1 (retrovirus) by Fugene HD (Promega, CA USA). After 48 hours, the supernatants were gathered and concentrated with Lenti-X/Retro-X solutions (Takara Bio USA Inc., CA USA). The lentivirus of CSII-CMV-mCherry-IRES-luciferase2 were transfected into the parental 143B cell line, and the subcloned cell line was finally named as 143B. 143B+PEDF cell line was made from 143B, and the additional infection of pDON-5-PEDF-HA was performed, and the clone was selected with puromycin and neomycin. Bkid cells were established as described in main figure 1E, and below. Then, to make PEDF-knockdown cells, the additional infection of pDON-5-PEDFmiR was performed, and we obtained Bkid+PEDFmiR cells used in this study.

**Retrieving metastatic cells from the tumors**

The metastatic lesions were dissected under a fluorescence dissecting microscope, dissociated with collagenase, and the dissociated cells were plated on collagen-coated dishes (IWAKI Co Ltd., Osaka, Japan). After attachment, the cells were rinsed, and puromycin was added into the culture medium. The cells derived from mouse tissues died after antibiotic treatment; however, the cancer cells survived because of the puromycin resistance gene in the CSII vector. We repeated the selection, the intracardiac injection, and monitored the metastasis toward various organs using *in vivo* luciferase assay. Sub-cell lines were established from single cell clones.

**CRISPR/PITCh genome editing**

The data set of each genome editing were predicted by PITCh designer v2.0 (<https://www.mls.sci.hiroshima-u.ac.jp/smg/PITChdesigner/index.html>). The oligonucleotides of the target sequences of Cas9 were synthesized and inserted into GeneArt® CRISPR Nuclease Vector Orange Fluorescent Protein reporter vector (Invitrogen). The oligonucleotide sequences were listed below. The procedure for constructing the working vectors followed the company's manual. PITCh methods were described in main text (13,14). The method is based on the microhology-mediated end joining (MHEJ), thus, the neighbor sequences of PAM sequence (shown as left/right homology arm in Supplemental Table-2) were used for the recombination of the target genome sequences with the target vectors. The cassete of CMV-GFP-P2A-Neomycin resistant gene in the donor vector was inserted into the target genome region. The edited clones were briefly screened by the genomic PCR (primers were described in Supplemental Table-2). And the clones of the potential candidates were analyzed by western blot.

**Supplemental Table-1 Quantitative PCR primers**

| Gene | Sequences (5’>3’) Upper | Lower |
| --- | --- | --- |
| hSLC14A1 | GAGGAATGTTCATGGCGCTC | ACAATAGCGTGGCCAAACAG |
| hLAMC2 | CATTAGACGGCCTCCTGCAT | CGCAGTTGGCTGTTGATCTG |
| hAmelotin | AGTCAGGCAGGGGCTAATCC | GGGTGGTCACTGCAAAGTCG |
| hDDIT4 | GGGAGGAGGGGTTTGACCG | CCCATCCAGGTAAGCCGTGT |
| hLURAP1 | GCTTCGAGGCCCACTGGTTC | GTGCAGCCTCTTCCTAGTCTCAG |
| hPEDF | AGCCTGCTGGACGCTGGATTA | CCAATGCAGAGGAGTAGCACCA |
| hSlitrk6 | ACACCTCTTCCAGCTGTGCAT | AGCACTGGAGTTTGGGAGTGT |
| hGAPDH | TGACATCAAGAAGGTGGTGAAGC | CACCCTGTTGCTGTAGCCAAAT |
| mGAPDH | ATGTGTCCGTCGTGGATCTG | ACCCTGTTGCTGTAGCCGTAT |
| mMMP2 | ACAAGTGGTCCGCGTAAAGT | AAACAAGGCTTCATGGGGGC |
| mSnail2 | CTCGGGAGCATACAGCCCTA | GCAGTCTCTCCTCTTCGTCAC |
| mLoxl2 | GCCATGCGTGGATTTGGCAC | ATCATAGCGGCTCCTGCACT |
| mSNAI3 | TCCAGGGTCACATCCGCACC | ATGCCGCGCCAAGAGAGACA |
| mSNAI3-3’UTR | GAGGGGCCAAACCCGACAATC | GGGGAAGATGCCCGAAGGC |
| mLoxl1 | TACTTGCCCGTGCGAAGCTC | CGCGACCATTCTGGTTGGGT |
| mTcf12 | ACTTCACTCCCTGCAGTCTCG | GGATGGTCCCAGCAAACTGTGT |
| mS100A4 | AGCACTTCCTCTCTCTTGGTCTGG | AGGCAGCTCCCTGGTCAGTAG |
| mACTA2 | GTACCACCATGTACCCAGGC | GAAGGTAGACAGCGAAGCCA |
| mSM-22a | TGGTTTATGAAGAAAGCCCAGGAG | GGTCGCCCATAGCCTGTCAT |
| mMTDH | GGAAGTTGATGAAGGAGCCTGGG | AGTTGCTCGGTGGTAACTGTGA |

Human primers were used in main Figure 2. Mouse primers were used for TKD2, mouse kidney endothelial cell line in Figure 6.

**Supplemental Table-2 Cloning Primers and CRISPR constructs**

| Item | Sequences (5’>3’) *Italic means artificial sequences* | | | | | |
| --- | --- | --- | --- | --- | --- | --- |
| PEDF_Fw1 | ATGCAGGCCCTGGTGCTAC | | | | | |
| PEDF_Rv1 | *ggtattggaatattaaactgggata*TTAGG | | | | | |
| PEDFHA_Rv2 | *gcacgtcataaggata*GGGGCCCCTGGGGTCCAG | | | | | |
| PEDF_Fw2 | *gagctcggatctcacgtgg*ATGCAGGCCCTGGTGCTACTC | | | | | |
| pDON5HAFw | *atatccttatgacgtgc*CTGACTATGCCTAAAACGCATGCATCTCAATTAGTC | | | | | |
| pDON5vecRv | CACGTGAGATCCGAGCTCGGTACCAAGCTTAAGTTTGG | | | | | |
| PEDFmiRa | TGCTGATCTCTTGCAGGTCCAAGCGAGTTTTGGCCACTGACTGACTCGCTTGGCTGCAAGAGAT | | | | | |
| PEDFmiRb | TGCTGCGAAAGGGTTCATCTCGGACGGTTTTGGCCACTGACTGACCGTCCGAGGAACCCTTTCT | | | | | |
| mLamR_Fw/Rv for ORF cloning | *tttcagagcgataacTCCGGAGCCCTTGACGTC* | | | *ccggatcagcttgcaTCAGGACCACTCAGTGGT* | | |
| mLamR_sgRNA_Fw/Rv | CCTTGACGTCCTGCAGATGAGTTTT | | | TCATCTGCAGGACGTCAAGGCGGTG | | |
| L/R homology arm_mLamR | GAGCCCTTGACGTCCTGCAGNNNNNNAAGGAGGAGGATGTCCTCAA | | | | | |
| mLamR genomic DNA primers | TATCCTGTAGTCAGTGTGCCTGC | | | | CACAAGCCAGAGTAGCACTCCAA | |
| hLamR_sgRNA_Fw/Rv | AGGGAAATTTTCACAATGTCGTTTT | GACATTGTGAAAATTTCCCTCGGTG | | | | |
| L/R homology arm_hLamR | CTTAAAGGGAAATTTTCACANNNNN NTCCGGAGCCCTTGATGTCCT | | | | | |
| hLamR genomic DNA primers | TTTTGGTTGCTTTTAAGGGTGTGC | | | | | GCAATGACTAACCATCACTTTTCCTTT |
| PITCh target_sgRNA | GCATCGTACGCGTACGTGTT | | | | | |
| mLoxl1-ORF_Fw/Rv | ATGGCTCTGGCCGGAGCCGGC | | TCAGGACTGGACGATTTTGCAGT | | | |
| mLoxl1∆Lox_Fw/Rv | *ttgaattcaATGGCTCTGGCCGGAGCCGGC* | | aagaattcttAGCGACCATTCTGGTTGGGT | | | |
| mSNAI3_Fw/Rv | *gagatctcATGCCGCGCTCCTTCCTGGT* | | CTAGGGGCCAGGACAGCAGCCGGCT | | | |

The oligonucleotides used for the construction of the overexpression, knockdown, or knockout vectors. The italic letters indicated the artificial sequences for tagging protein and making the constructs. The oligonucleotides for microRNAs were inserted into pcDNA6.2 Gateway Block-IT miR vector (Invitrogen). The oligos for sgRNAs were boiled and annealed each other, and the double strand DNA fragments were inserted into GeneArt® CRISPR nuclease vector. The PITCh donor vector was made from the oligonucleotides encoding the left and the right homology sequences shown above.

**2. Supplemental Figures**


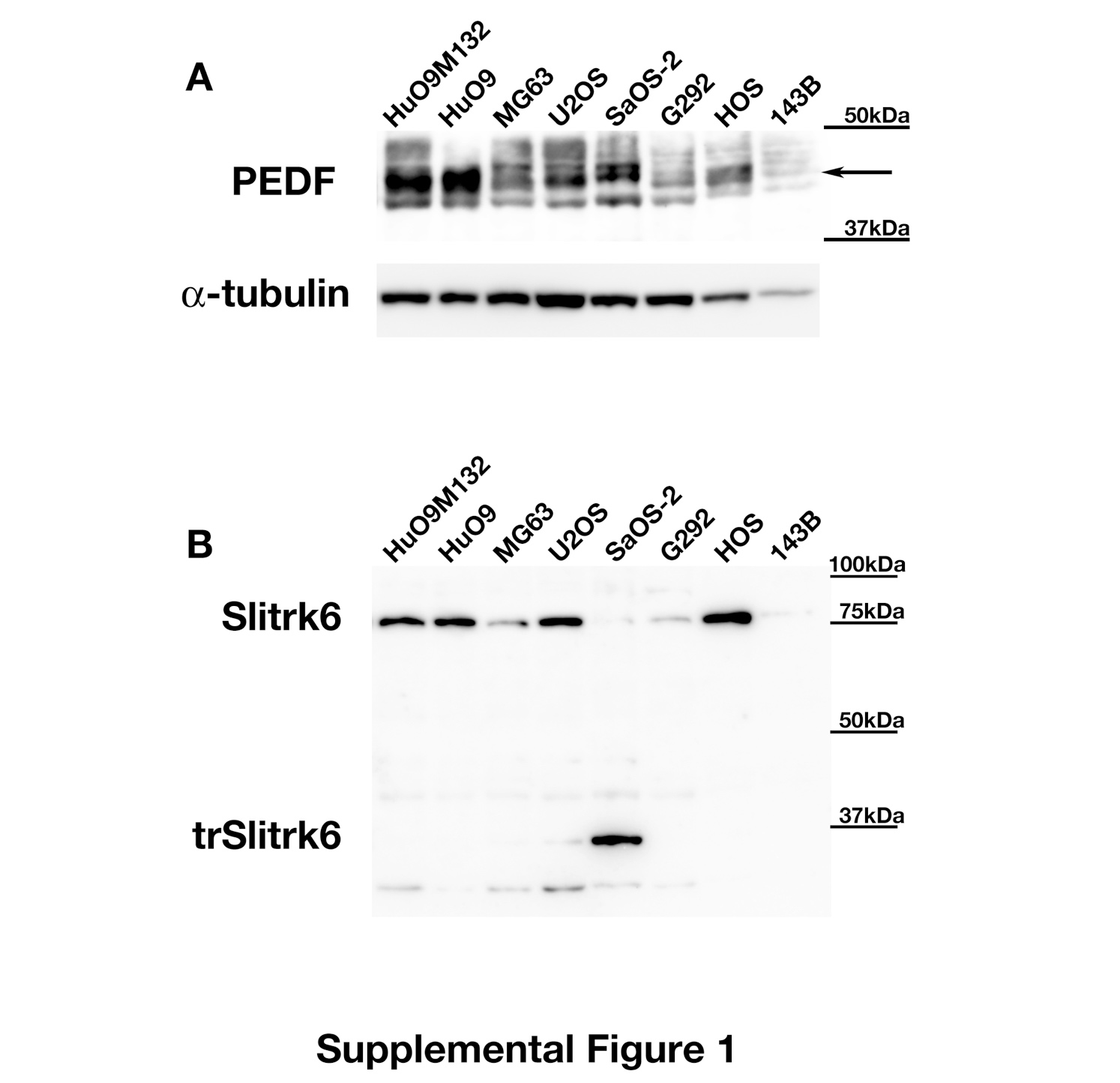


**Supplementary Figure 1.** **The gene profiles of PEDF and Slitrk6 in osteosarcoma**

(A) Immunoblot of osteosarcoma cell lysates by anti-PEDF antibody and anti-alpha-tubulin antibody. HuO9M132 is a highly metastatic mutant of HuO9. (B) The same membrane as above rehybridized by anti-Slitrk6 antibody. Slitrk6 in SaOS-2 was truncated. α-tubulin blot is common for (A) and (B).

**
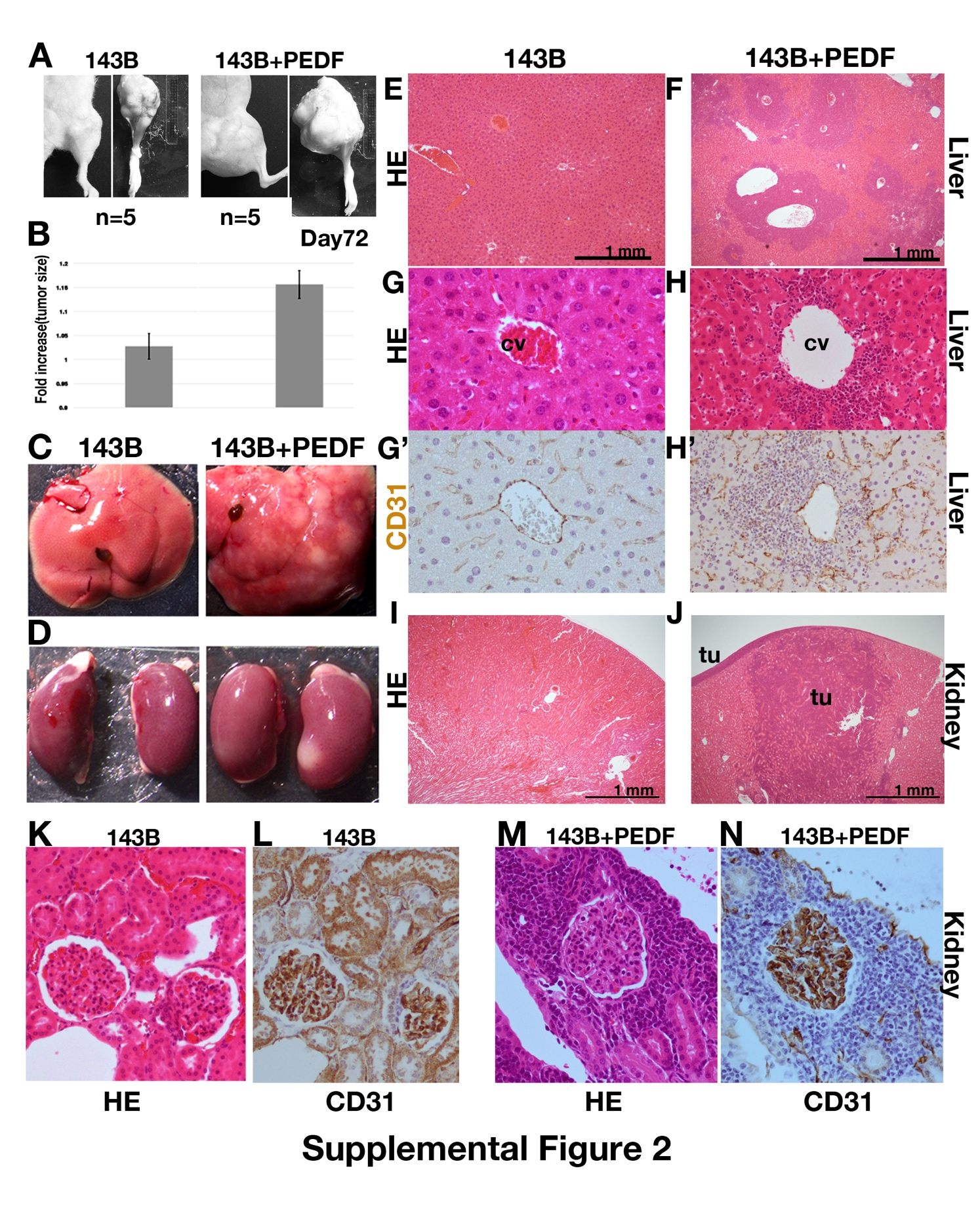
**

**Supplemental Figure 2 Reproduction of renal-metastatic phenotypes from the orthotopic injection**

(A-N) The results of low dose 143B/ 143B+PEDF cells (5 x 10^4^ cells) in the knee joint of the nude mouse. (A) The tumors after 72 days from the 143B cell injections or 143B+PEDF-injected mouse. (B) The fold increase of the average diameters of the tumor. (C) liver (D) kidneys of 143B or 143B+PEDF-injected mice. (E-H’) The liver section with H&E staining or (C). (E-H) H&E staining of the liver sections. (E) The normal liver in 143B-injected mouse. (F) Many inflammations were seen in 143B+PEDF-injected mouse. (G) The normal liver tissue in 143B-injected mouse. (H) The inflammatory cells were accumulated around the vein. (G’) CD31-IHC of the normal liver taken from 143B-injected mice. (H’) CD31-IHC of the inflammatory liver tissues in 143B+PEDF-injected mouse. (I-N) The section of the kidney in 143B or 143B+PEDF-injected mouse. (I) 143B-injected mouse kidney. No tumor was observed. (J) The section of the kidney in 143B+PEDF-injected mouse. The cancer cells were infiltrated into the kidney stroma. The tumor was also seen on the cortical area. (K) Normal kidney H&E staining from 143B-injected mouse. (L) CD31-IHC of the kidney in 143B-injected mouse. The glomerulus was strongly stained. (M) H&E staining of the kidney in 143B+PEDF-injected mouse. (N) CD31-IHC on the similar section to (M). The cancer cells were surrounded around the glomerulus.

**
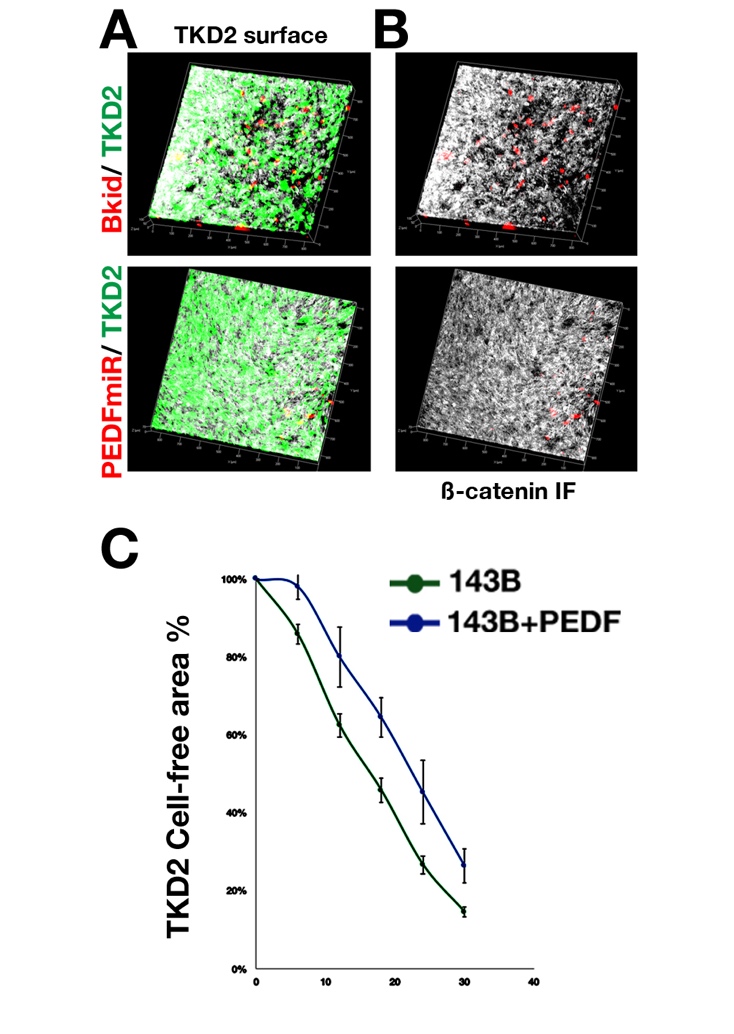
**

**Supplemental Figure 3 PEDF disturbed kidney endothelial cell layer formation**

(A) The similar assay described in Figure 6E. Bkid and TKD2-GFP cells were co-cultured on each side of the atterocollagen membrane culture insert (above). Bkid+PEDFmiR and TKD2GFP cells were co-cultured on each side of the atterocollagen membrane culture insert (below). (B) ß-catenin immune fluorescence (IF) of (A). TKD2 layer around the Bkid cells (red) were disturbed (above). Bkid+PEDFmiR cells were gone during the staining procedure because of the poor cell adhesion (below). (C) Wound healing assay performed similarly to Figure 6H. 143B or 143B+PEDF were cultured with TKD2 cell layer. 143B+PEDF cells delayed the cell migration of TKD2.
